# The epidemiological burden of obesity in childhood: a worldwide epidemic requiring urgent action

**DOI:** 10.1186/s12916-019-1449-8

**Published:** 2019-11-25

**Authors:** Mariachiara Di Cesare, Maroje Sorić, Pascal Bovet, J Jaime Miranda, Zulfiqar Bhutta, Gretchen A Stevens, Avula Laxmaiah, Andre-Pascal Kengne, James Bentham

**Affiliations:** 1 Middlesex University, The Burroughs, London, NW4 4BT UK; 2 University of Zagreb, Trg Republike Hrvatske 14, 10000 Zagreb, Croatia; 3 University of Ljubljana, Kongresni trg 12, 1000 Ljubljana, Slovenia; 4 Center for Primary Care and Public Health, Secteur Croisettes/Bâtiment SC-B, Route de la Corniche 2, 1066 Epalinges, Lausanne, Switzerland; 5 Ministry of Health, Hospital Road, Victoria, Republic of Seychelles; 6 Universidad Peruana Cayetano Heredia, Av. Honorio Delgado 430, San Martín de Porres, 15102 Lima, Peru; 7 Aga Khan University, National Stadium Rd, Karachi, 74800 Pakistan; 8 Independent consultant, Los Angeles, USA; 9 c/o: School of Mathematics, Statistics and Actuarial Science, University of Kent, Giles Lane, Canterbury, CT2 7NZ UK; 10 ICMR-National Institute of Nutrition, Beside Tarnaka Metro Station, Osmania University PO, Hyderabad, Telangana 500007 India; 11 South African Medical Research Council, Francie Van Zijl Dr, Parow Valley, Cape Town, 7501 South Africa; 12 University of Kent, Giles Lane, Canterbury, CT2 7NZ UK

**Keywords:** Obesity, Overweight, Global health, Children, Adolescents

## Abstract

**Background:**

In recent decades, the prevalence of obesity in children has increased dramatically. This worldwide epidemic has important consequences, including psychiatric, psychological and psychosocial disorders in childhood and increased risk of developing non-communicable diseases (NCDs) later in life. Treatment of obesity is difficult and children with excess weight are likely to become adults with obesity. These trends have led member states of the World Health Organization (WHO) to endorse a target of no increase in obesity in childhood by 2025.

**Main body:**

Estimates of overweight in children aged under 5 years are available jointly from the United Nations Children’s Fund (UNICEF), WHO and the World Bank. The Institute for Health Metrics and Evaluation (IHME) has published country-level estimates of obesity in children aged 2–4 years. For children aged 5–19 years, obesity estimates are available from the NCD Risk Factor Collaboration. The global prevalence of overweight in children aged 5 years or under has increased modestly, but with heterogeneous trends in low and middle-income regions, while the prevalence of obesity in children aged 2–4 years has increased moderately. In 1975, obesity in children aged 5–19 years was relatively rare, but was much more common in 2016.

**Conclusions:**

It is recognised that the key drivers of this epidemic form an obesogenic environment, which includes changing food systems and reduced physical activity. Although cost-effective interventions such as WHO ‘best buys’ have been identified, political will and implementation have so far been limited. There is therefore a need to implement effective programmes and policies in multiple sectors to address overnutrition, undernutrition, mobility and physical activity. To be successful, the obesity epidemic must be a political priority, with these issues addressed both locally and globally. Work by governments, civil society, private corporations and other key stakeholders must be coordinated.

## Background

Excess weight during childhood and adolescence remains one of the most important issues in global health, despite emerging as a concern several decades ago [1, 2]. Recent estimates suggest that 40 million children under the age of 5 years and more than 330 million children and adolescents aged 5–19 years were overweight or obese in 2016 [3]. Given the global emergency posed by excess weight in children, member states of the World Health Organization (WHO) endorsed “no increase in childhood overweight by 2025” as one of the six global nutrition targets in the ‘Comprehensive Implementation Plan for Maternal, Infant and Young Child Nutrition’ [4]. This is consistent with the same target for obesity and diabetes between 2010 and 2025 in the ‘WHO Global Action Plan for the Prevention and Control of Non-communicable Diseases 2013–2020’ [5, 6].

Overweight or obesity during childhood has important short-term and long-term consequences. In the short term, children who are overweight or obese are more likely to suffer from psychological comorbidities such as depression, anxiety, low self-esteem, a series of emotional and behavioural disorders [7, 8], asthma [9], low-grade systemic inflammation [10, 11], liver complications [12, 13], and musculoskeletal problems, especially in the lower extremities [14]. Children who are overweight and obese also have more metabolic and cardiovascular risk factors [15, 16], such as high blood pressure [17], dyslipidaemia [18], type 2 diabetes [19] and other abnormalities of the cardiovascular system [20]. In the long term, overweight or obesity during childhood increases the risk of developing cardiovascular diseases, diabetes, some cancers, and musculoskeletal disorders in adulthood, which can lead to disability [21] and premature death [22–24]. In addition, the treatment of obesity in adulthood is difficult [25], with evidence suggesting that around three-quarters of children who are overweight or obese carry this status into adulthood [26]. Strong persistence of overweight status and low efficacy of available treatments highlight the need to prevent overweight and obesity at the earliest possible stage of life.

It is recognised that weight gain is partly caused by elevated energy intake, which often includes a disproportionate amount of refined carbohydrates and/or processed foods (increasing insulin release and fat storage), and decreased physical activity [27]. Weight gain is also promoted by environmental, behavioural, biological, and genetic factors, whose interactions have driven the current levels of worldwide obesity. Maternal health status during pregnancy, an obese intrauterine environment [28] and rapid changes in weight status during infancy [29] are other factors contributing to obesity in children. Moreover, the expanding ‘obesogenic’ environment increases the propensity of children to consume foods and beverages that are high in calories, energy-dense, or low in nutrients, as well as promoting sedentary lifestyles through reductions in opportunities for active mobility in daily lives [30]. Key drivers of the rapidly increasing worldwide occurrence of obesity and diabetes across populations are the globalised market and commercial interests that favour the production and distribution of inexpensive, energy-dense foods and beverages and limited political will to address the economic causes of the obesity epidemic [3], which include a strong association with socioeconomic inequalities [31, 32]. In high-income settings, higher prevalence of obesity is observed in disadvantaged and marginalised communities than in groups with higher socioeconomic status [33–35]. In contrast, higher prevalence of obesity is seen in groups with higher socioeconomic status in some, but not all, low and middle-income settings [31].

Over the past decade, genome-wide association studies have been used to identify genetic markers that increase predisposition to weight gain, with the goal of explaining the biological mechanisms leading to obesity. For example, the *FTO* gene is recognised as being key to the regulation of energy intake, with variants predisposing individuals to greater caloric intake and reduced feelings of satiety [36]. Genetic and epigenetic factors also produce heterogeneity in obesity phenotypes across populations, including characteristic metabolic profiles and greater central body adiposity in south Asians [37]. However, groups with almost identical genotypes can have very different obesity phenotypes, as shown by the large differences in prevalence between Samoa and American Samoa [38]. In addition, obesity-associated genes cannot explain the rapid onset and scale of the current obesity epidemic, even if genetic predisposition makes some individuals more susceptible to the obesogenic environment [39].

Finally, obesity in childhood has important economic and social costs, with increased burdens on health systems as well as later reduced economic productivity [40–43]. For example, in the USA, the estimated direct medical cost over the lifetime of a 10-year-old child with obesity, compared with a similar child with normal weight and allowing for weight gain in adulthood, is between US$12,660 and US$19,630 [44].

Over the past decade, there has been a global effort to provide reliable and detailed estimates of the worldwide epidemic of excess weight in children and adolescents. Here, we aim to provide a comprehensive description of this work, presenting global, regional and national trends based on the most up-to-date information available. To do so, we use data from the United Nations Children’s Fund (UNICEF)/WHO/World Bank Joint Child Malnutrition Estimates [45], the Institute for Health Metrics and Evaluation (IHME) Global Burden of Disease Study [46] and the Non-Communicable Disease (NCD) Risk Factor Collaboration (NCD-RisC) [47] (see Table 1). It should be noted that there are two definitions of obesity in childhood: the International Obesity Taskforce (IOTF) definition [48] and one based on the WHO growth reference curve [49]. They have different age-specific cut-offs and can therefore give different obesity estimates for a given set of data. In the following, estimates published by IHME use the IOTF definition, while estimates published by UNICEF/WHO/World Bank and NCD-RisC use the WHO growth reference. Readers interested in the differences in statistical models and regional definitions in these studies are referred to the original papers.

**Table 1:** Definitions of overweight and obesity in different studies

Metric	Age group	Source	Definition	Years
Overweight	Under 5 years	UNICEF, WHO, World Bank [45]	WHO Child Growth Standard	1990–2018
Obesity	2–4 years	IHME [46]	International Obesity Task Force	1980–2015
Obesity	5–19 years	NCD-RisC [47]	WHO Growth Reference	1975–2016

*Abbreviations*: *IHME* Institute for Health Metrics, *NCD-RisC* Non-Communicable Disease Risk Factor Collaboration, *UNICEF* United Nations Children’s Fund, *WHO* World Health Organization

### Children aged 5 years or under

#### Global and regional trends in overweight

The most recent estimates of trends in overweight for children under the age of 5 years were published jointly by UNICEF, WHO and the World Bank in April 2019 [45]. Globally, the prevalence of overweight rose modestly, from 4.8% in 1990 to 5.9% in 2018, but with estimates for low and middle-income United Nations regions showing heterogeneous trends. Estimates were not published for high-income regions.

Table 2 presents results by region. In Africa as a whole, overweight prevalence changed little between 1990 and 2018. However, prevalence increased in Northern and Southern Africa, and also rose modestly in Middle Africa. This was offset by decreases in overweight prevalence in Eastern and Western Africa. Overweight prevalence in Asia rose, with increases in every region except Eastern Asia, where overweight prevalence remained almost unchanged. In Latin America and the Caribbean, overweight prevalence increased, including a moderate increase in the Caribbean and small increases in Central and South America. Finally, the overweight epidemic in Oceania (excluding Australia and New Zealand) became much more severe, with a three-fold increase in prevalence.

**Table 2:** Estimates of the proportion of overweight children under 5 years of age, by region

Region	Estimated overweight proportion in 1990 (%) (uncertainty interval)	Estimated overweight proportion (%) in 2018 (uncertainty interval)
**Africa**	**5.2 (4.2–6.2)**	**4.9 (3.6–6.1)**
Northern Africa	7.0 (4.2–11.4)	10.6 (4.8–21.8)
Middle Africa	4.1 (2.2–7.5)	4.6 (3.4–6.3)
Southern Africa	9.1 (6.4–12.9)	13.0 (9.3–17.9)
Eastern Africa	5.0 (3.7–6.7)	4.3 (3.5–5.3)
Western Africa	3.9 (2.8–5.5)	2.1 (1.6–2.6)
**Asia**	**4.0 (3.0–5.0)**	**5.2 (3.9–6.5)**
Eastern Asia	6.4 (5.8–7.0)	6.3 (5.5–7.2)
Western Asia	5.7 (3.0–10.5)	9.0 (3.5–21.1)
Southern Asia	2.3 (0.8–6.1)	3.1 (1.9–5.1)
South-eastern Asia	1.9 (1.5–2.4)	7.7 (4.0–14.2)
Oceania (excluding Australia and New Zealand)	3.2 (2.4–4.2)	9.1 (5.9–13.8)
**Latin America and the Caribbean**	**6.2 (4.7–7.7)**	**7.5 (6.6–8.4)**
Caribbean	4.2 (4.0–4.5)	7.0 (3.7–12.8)
Central America	5.3 (3.7–7.6)	6.9 (6.0–8.1)
South America	6.8 (4.9–9.3)	7.8 (6.7–9.1)

Estimates were published jointly by the United Nations Children’s Fund (UNICEF), the World Health Organization and the World Bank (see Table 1)

Bold shows regional aggregates

#### Trends in obesity prevalence

**Fig. 1:**
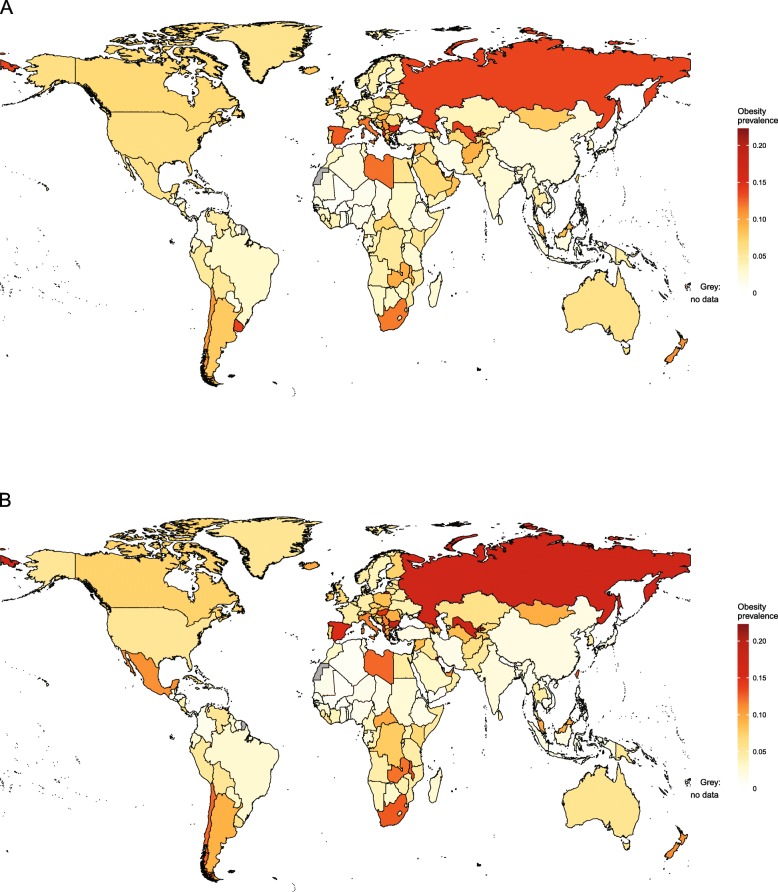
Obesity prevalence for girls and boys aged 2–4 years in 1980, by country. Estimates of obesity prevalence in (**a**) girls and (**b**) boys aged 2–4 years were published by the Institute for Health Metrics and Evaluation using the International Obesity Taskforce growth reference [46] (see Table 1)

**Fig. 2:**
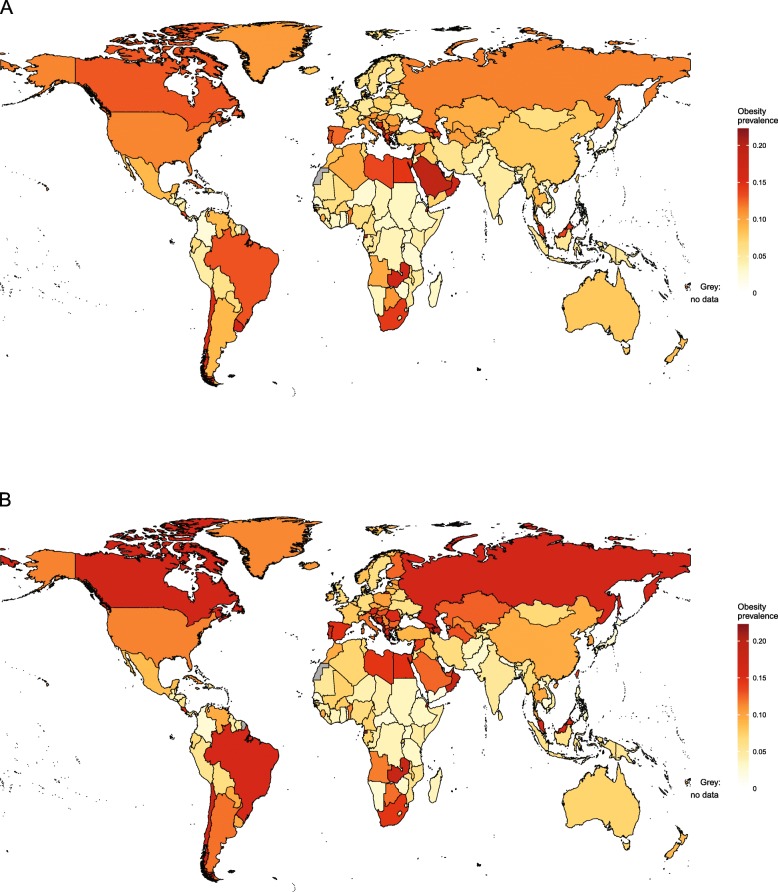
Obesity prevalence for girls and boys aged 2–4 years in 2015, by country. Estimates of obesity prevalence in (**a**) girls and (**b**) boys aged 2–4 years were published by the Institute for Health Metrics and Evaluation using the International Obesity Taskforce growth reference [46] (see Table 1)

Obesity trends in children aged 2–4 years are available from IHME for the period 1980–2015 [46] and are the only source of comparable country-level information for children under the age of 5 years. National-level estimates for 1980 and 2015 are shown in Figs. 1 and 2, respectively.

At the global level between 1980 and 2015, the prevalence of obesity increased from 3.9 to 7.2% in boys and from 3.7 to 6.4% in girls aged 2–4 years. In 2015, by far the highest levels of obesity were in American Samoa, where around 50% of girls and boys in this age group were obese. More than one in three girls were obese in Kiribati and more than one in four in Samoa and Kuwait. For boys, the second highest prevalence of obesity in this age group was in Kuwait, followed by Qatar and Kiribati. For girls, the lowest prevalence of obesity was seen in North Korea, followed by Eritrea, Bangladesh and Burundi. In boys, the lowest prevalence was in Eritrea, followed by North Korea, Burundi and Bangladesh.

Table 3 presents estimates by region. As shown in Figs. 1 and 2, patterns are heterogeneous in sub-Saharan Africa. In 1980, obesity was most common in girls and boys in South Africa and least common in girls and boys in Mali. By 2015, the country with the highest prevalence of obesity in girls was Equatorial Guinea, followed by Djibouti, Zambia and South Africa. Countries with the highest prevalence of obesity in boys were also Equatorial Guinea, followed by Zambia, Djibouti and South Africa. In contrast, less than 2% of girls in Eritrea and Burundi and less than 1% of boys in Eritrea were obese.

**Table 3:** Estimates of the proportion of obese children aged 2–4 years, by region

Region	Sex	Estimated proportion of obese children in 1980 (%) (uncertainty interval)	Estimated proportion of obese children in 2015 (%) (uncertainty interval)
Sub-Saharan Africa	Female	3.7 (3.1–4.4)	5.4 (4.5–6.5)
	Male	4.3 (3.4–5.5)	5.8 (4.7–7.1)
South Asia	Female	2.8 (1.5–4.9)	4.0 (2.1–7.1)
	Male	2.4 (1.3–4.2)	4.5 (2.3–8.1)
East and Southeast Asia and Oceania	Female	2.3 (1.4–3.8)	6.8 (4.2–10.8)
	Male	2.3 (1.4–3.7)	8.1 (4.8–12.5)
High-income countries	Female	6.0 (4.9–7.2)	8.9 (7.2–10.9)
	Male	6.1 (4.9–7.6)	10.0 (8.0–12.4)
Latin America and the Caribbean	Female	3.9 (2.7–5.7)	8.7 (6.0–12.4)
	Male	5.0 (3.2–8.0)	9.8 (6.4–14.1)
Middle East and North Africa	Female	4.3 (3.7–5.1)	9.2 (7.6–10.9)
	Male	3.5 (2.9–4.3)	8.8 (7.3–10.7)
Central and Eastern Europe and Central Asia	Female	9.0 (7.3–11.1)	9.3 (7.7–11.3)
	Male	11.5 (9.4–14.1)	12.6 (10.4–15.0)

Estimates were published by the Institute for Health Metrics and Evaluation (see Table 1)

In south Asia in 1980, the prevalence of obesity was highest in girls and boys in Afghanistan and lowest in girls and boys in Nepal (Fig. 1). By 2015, the highest prevalence of obesity was seen in Bhutan and the lowest in Bangladesh for both sexes (Fig. 2). In 1980 in East and Southeast Asia, obesity prevalence was highest in girls in Malaysia and boys in Taiwan and was lowest in girls in the Philippines and boys in Vietnam. In 2015, the highest levels of obesity in girls were seen in Malaysia, followed by Thailand and China, while in boys, the highest obesity was also seen in Malaysia, followed by Taiwan and Thailand. North Korea had the lowest level of obesity for both sexes.

Obesity prevalence in children aged 2–4 years was heterogeneous in Oceania. In 1980, while almost half of girls and boys in American Samoa were obese, this was the case for fewer than 1 in 20 girls in Papua New Guinea and boys in Fiji. In 2015, obesity ranged from approximately 50% in American Samoa to around 5% in Papua New Guinea in both sexes.

In Latin America and the Caribbean in 1980, the highest levels of obesity were seen in girls in Uruguay and boys in Chile. The lowest levels of obesity were seen in girls in Colombia and boys in Honduras. By 2015, the highest levels of obesity were seen in Puerto Rico for both girls and boys. For girls, the next highest levels of obesity were seen in Dominica and Uruguay, while for boys, Puerto Rico was followed by Chile and Barbados. The lowest prevalence of obesity was seen in Haiti and Colombia for both boys and girls.

In the Middle East in 1980, the highest levels of obesity were seen in girls in Kuwait and boys in Qatar, while the lowest levels were seen in girls in Iran and boys in Yemen. By 2015, the highest levels of obesity were seen in girls in Kuwait, Saudi Arabia and Qatar and in boys, in Kuwait, Qatar and Oman. This contrasted with girls in Jordan and boys in Yemen, for whom obesity rates were lowest. In North Africa in 1980, the highest prevalence of obesity was seen in girls and boys in Libya and the lowest prevalence was in girls and boys in Algeria. By 2015, the highest obesity prevalence was seen in girls and boys in Egypt, while the lowest level was in Tunisia for both sexes.

In high-income countries, obesity prevalence increased between 1980 and 2015 (Figs. 1 and 2). In high-income Western countries in 1980, the highest obesity prevalence was in girls in Andorra and boys in Spain, with the lowest levels of obesity in girls in Switzerland and boys in the Netherlands. In 2015, the highest levels of obesity in girls were still in Andorra, followed by Malta, Greece and Portugal. In boys, the highest levels were in Luxembourg, Andorra, Canada and Malta. The lowest levels were in girls and boys in Switzerland. In high-income Asia-Pacific, the highest obesity prevalence in 1980 was seen in girls and boys in Singapore and the lowest in girls and boys in Japan. By 2015, obesity prevalence exceeded 10% in boys and 6% in girls in Singapore and South Korea. In contrast, obesity prevalence was less than 3% in girls and boys in Japan.

In Central and Eastern Europe in 1980, the highest obesity prevalence was seen in girls in Albania and boys in Bulgaria, with the lowest in girls and boys in Ukraine. In 2015, obesity was particularly high in girls in Albania, followed by Montenegro, Bosnia and Herzegovina and Russia. Albania also had the highest prevalence of obesity in boys, followed by Montenegro, Russia and Bosnia and Herzegovina. Obesity prevalence was lowest in girls in Ukraine, followed by Moldova, while in boys, the lowest obesity was in Moldova, followed by Ukraine. In Central Asia in 1980, obesity was most common in girls and boys in Uzbekistan and least common in girls and boys in Kazakhstan. In 2015, the prevalence of obesity was highest in girls in Georgia and boys in Azerbaijan and lowest in both sexes in Kyrgyzstan.

**Fig. 3:**
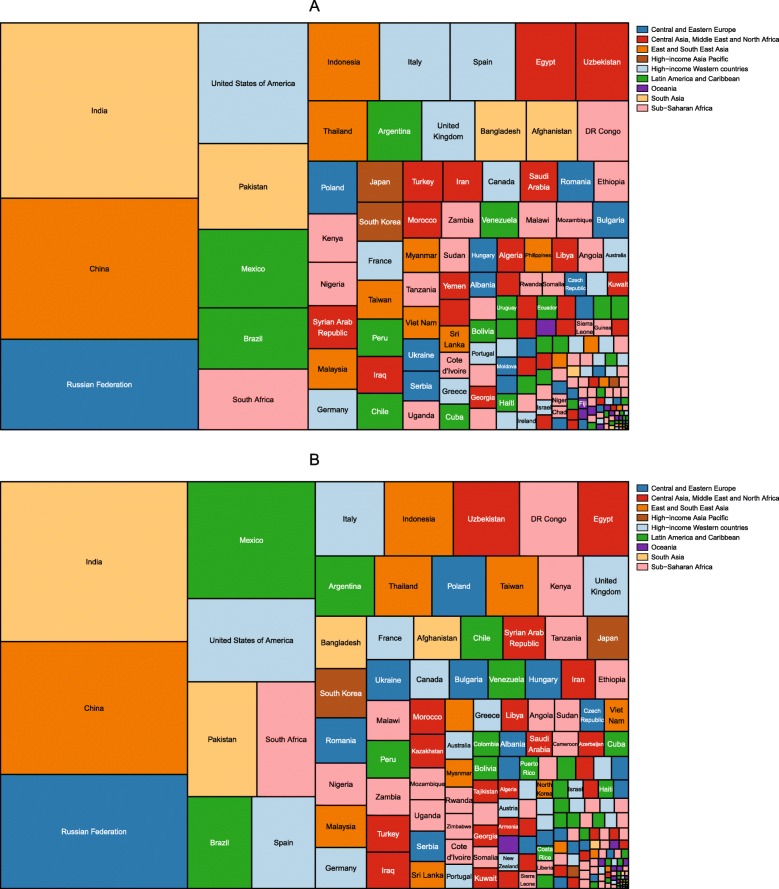
Division of the number of girls and boys aged 2–4 years with obesity in 1980, by country. Estimates of obesity in (**a**) girls and (**b**) boys were published by the Institute for Health Metrics using the International Obesity Taskforce growth reference [46] (see Table 1)

**Fig. 4:**
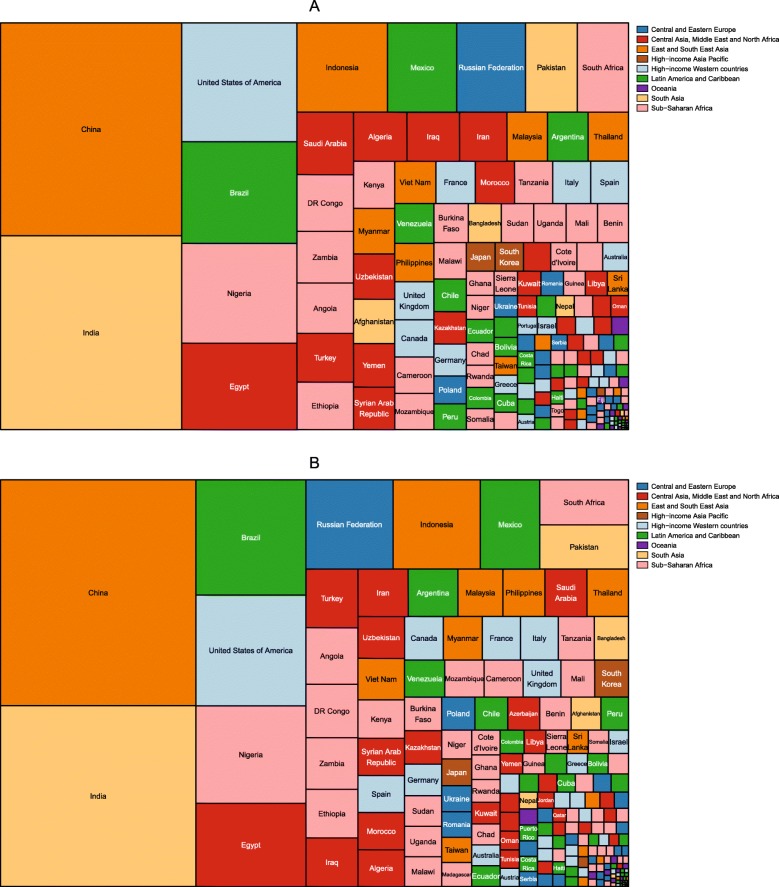
Division of the number of girls and boys aged 2–4 years with obesity in 2015, by country. Estimates of obesity in (**a**) girls and (**b**) boys were published by the Institute for Health Metrics using the International Obesity Taskforce growth reference [46] (see Table 1)

The number of children aged 2–4 years with obesity was also published by IHME for the period 1980–2015 [46]. The division of these children by country in 1980 and 2015 is shown in Figs. 3 and 4, respectively. In 1980, the country with the largest number of girls with obesity was India, followed by China, Russia and the USA. India, China and Russia also had the largest number of boys with obesity, followed by Mexico. By 2015, China had the largest number of girls with obesity, followed by India, the USA and Brazil. The largest number of boys with obesity was in China, followed by India, Brazil and the USA.

### Children and adolescents aged 5–19 years

#### Worldwide trends in obesity

**Fig. 5:**
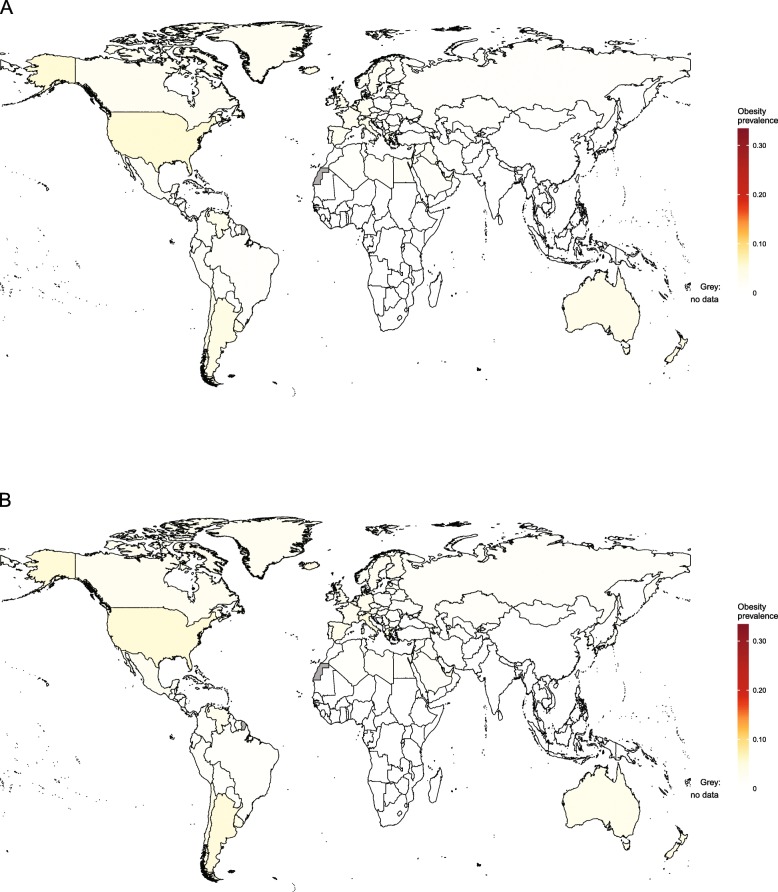
Obesity prevalence for girls and boys aged 5–19 years in 1975, by country. Estimates of obesity in (**a**) girls and (**b**) boys were published by the Non-Communicable Diseases Risk Factor Collaboration (NCD-RisC) using the World Health Organization growth reference [47] (see Table 1)

**Fig. 6:**
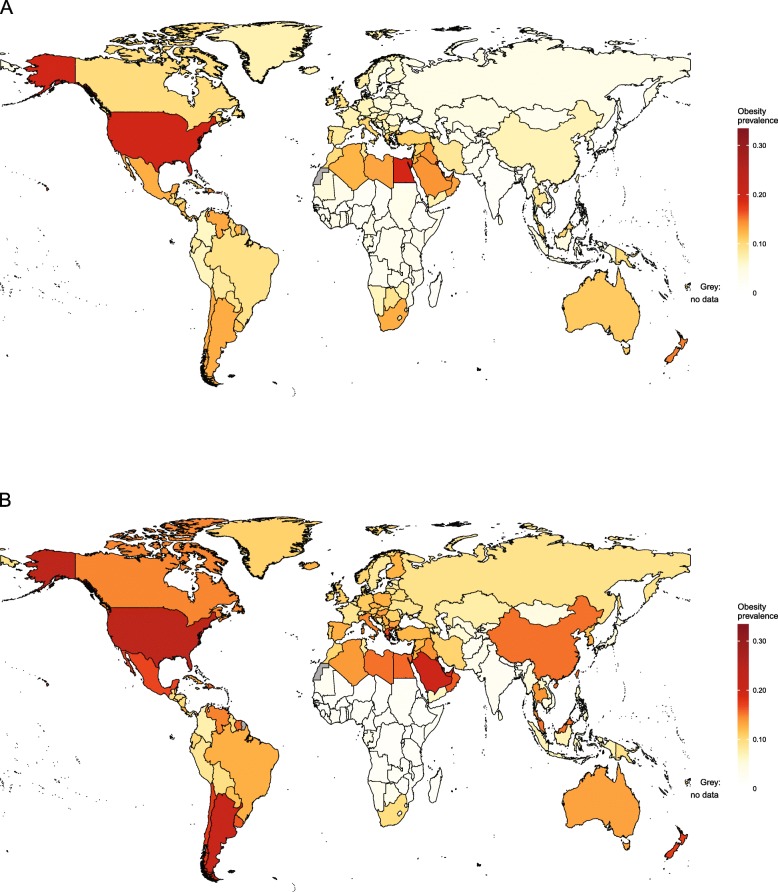
Obesity prevalence for girls and boys aged 5–19 years in 2016, by country. Estimates of obesity in (**a**) girls and (**b**) boys were published by the Non-Communicable Diseases Risk Factor Collaboration (NCD-RisC) using the World Health Organization growth reference [47] (see Table 1)

NCD-RisC holds the largest global database on obesity in children and adolescents aged 5–19 [50]. The most recent estimates, published in 2017, were based on 2416 measured data sources [47]. They showed that between 1975 and 2016, obesity prevalence increased from 0.7 to 5.6% in girls and from 0.9 to 7.8% in boys. However, the global increase in obesity masked heterogeneous trends at national level, as shown in Figs. 5 and 6.

#### Obesity trends by region

Table 4 presents results by region. As shown in Fig. 5, obesity was rare across the world in 1975, but particularly so in sub-Saharan Africa, with an estimated prevalence of 0.1% for girls and 0.0% for boys. An obesity prevalence greater than 0.5% was observed only in Djibouti and Seychelles for girls and in Seychelles for boys. By 2016, an obesity prevalence of greater than 5% was seen in 10 countries for girls and two for boys (Fig. 6). Six of the seven countries with the highest rates of obesity in girls were in southern Africa, with South Africa having the highest prevalence and Burkina Faso the lowest prevalence. For boys, Seychelles had the highest prevalence, followed by South Africa and with Uganda having the lowest prevalence.

**Table 4:** Estimates of the proportion of obese children and adolescents aged 5–19 years, by region, published by the Non-Communicable Diseases Risk Factor Collaboration (NCD-RisC; see Table 1)

Region	Sex	Estimated % obese children and adolescents in 1975 (uncertainty interval)	Estimated % obese children and adolescents in 2016 (uncertainty interval)
Sub-Saharan Africa	Female	0.1 (0.0–0.3)	3.2 (2.2–4.4)
	Male	0.0 (0.0–0.2)	1.7 (0.9–2.8)
South Asia	Female	0.0 (0.0–0.1)	1.8 (1.0–3.1)
	Male	0.0 (0.0–0.2)	2.6 (1.4–4.5)
East and Southeast Asia	Female	0.1 (0.0–0.2)	5.9 (4.2–8.1)
	Male	0.2 (0.0–0.7)	12.1 (9.0–15.7)
High-income Asia-Pacific	Female	0.5 (0.3–0.9)	2.7 (2.0–3.6)
	Male	1.5 (0.8–2.8)	7.5 (5.9–9.3)
Latin America and the Caribbean	Female	1.6 (0.4–3.8)	10.4 (7.8–13.6)
	Male	1.8 (0.3–4.9)	13.4 (10.0–17.2)
North Africa, the Middle East and Central Asia	Female	0.9 (0.1–2.5)	11.3 (8.1–15.1)
	Male	0.8 (0.1–2.8)	12.0 (8.6–15.9)
High-income Western countries	Female	3.8 (2.1–6.1)	13.3 (10.3–16.6)
	Male	4.1 (2.3–6.4)	16.8 (13.2–20.6)
Central and Eastern Europe	Female	0.8 (0.1–2.8)	5.0 (2.8–8.3)
	Male	1.1 (0.1–3.5)	10.1 (6.2–15.6)
Oceania	Female	0.7 (0.1–3.9)	10.7 (4.2–21.0)
	Male	0.7 (0.0–3.7)	9.9 (3.5–20.5)

South Asia also had extremely low levels of obesity in 1975, estimated at 0.0% for both girls and boys and reaching a maximum of 0.1% for boys in Pakistan. However, obesity was less rare by 2016, with Afghanistan for girls and Bhutan, Pakistan and Bangladesh for boys having a prevalence of obesity of greater than 3%. More heterogeneous trends were observed in East and Southeast Asia. In 1975, obesity in boys and girls was most common in Hong Kong, but the prevalence of obesity was less than 2% elsewhere in the region. In 2016, the highest level of obesity in girls was seen in Malaysia and the lowest in Cambodia. For boys, the prevalence of obesity was highest in Brunei Darussalam and lowest in Vietnam. Meanwhile, in high-income countries in Asia-Pacific in 1975, obesity prevalence was highest in Singapore for girls and boys. By 2016, the highest prevalence of obesity was in South Korea and the lowest in Japan for both sexes.

In 1975, obesity levels were low in Latin America and the Caribbean (Fig. 5). Obesity was most common in Bermuda, followed by Argentina and Uruguay for both sexes. By 2016, the prevalence of obesity had become more heterogeneous. For girls, the highest levels of obesity were seen in Puerto Rico, Bermuda and the Bahamas, while for boys the highest levels were seen in Bermuda, Argentina and Puerto Rico. Obesity prevalence was lowest in Colombia for girls and boys, followed by Peru and Haiti for girls and Saint Lucia and Peru for boys.

Heterogeneous trends were observed across North Africa, the Middle East and Central Asia. In 1975, obesity prevalence was highest in girls and boys in Kuwait. By 2016, obesity prevalence was highest in Kuwait and Egypt for girls and in Kuwait and Qatar for boys. Meanwhile, obesity prevalence was lowest in both sexes in Tajikistan.

There were heterogeneous patterns of obesity in high-income Western countries in both 1975 and 2016. In 1975, the highest level of obesity was in Malta for girls and boys, followed by the USA, Andorra and Israel for girls and Andorra, Israel and the USA for boys. Meanwhile, the prevalence of obesity was below 2% in eight countries for girls and in five countries for boys. By 2016, the highest levels of obesity were observed mostly in English-speaking and Mediterranean countries. The USA had the highest prevalence of obesity for girls and boys, followed by New Zealand. Switzerland had the lowest obesity prevalence among girls and boys.

In 1975, for both sexes, obesity prevalence was less than 2% in every country in Central and Eastern Europe (Fig. 5). By 2016, the prevalence of obesity had exceeded 13% in boys and 7% in girls in Croatia, Hungary and Bulgaria. Obesity prevalence was lowest among boys in Moldova, followed by Bosnia and Herzegovina and the three Baltic states. For girls, Moldova, Russia and Estonia had the lowest prevalence of obesity.

Obesity was uncommon in children and adolescents aged 5–19 years in Oceania in 1975, with prevalence exceeding 5% only in girls and boys in Nauru and in girls in Palau. By 2016, the 13 countries with the highest obesity rates for girls and the eight countries with the highest obesity rates for boys were all in Oceania; more than 30% of girls and boys in Nauru, the Cook Islands and Palau were obese. However, there was a contrast between patterns in Melanesia, and Polynesia and Micronesia, with obesity prevalence lower in all countries in Melanesia.

#### Changes in obesity at national level

**Fig. 7:**
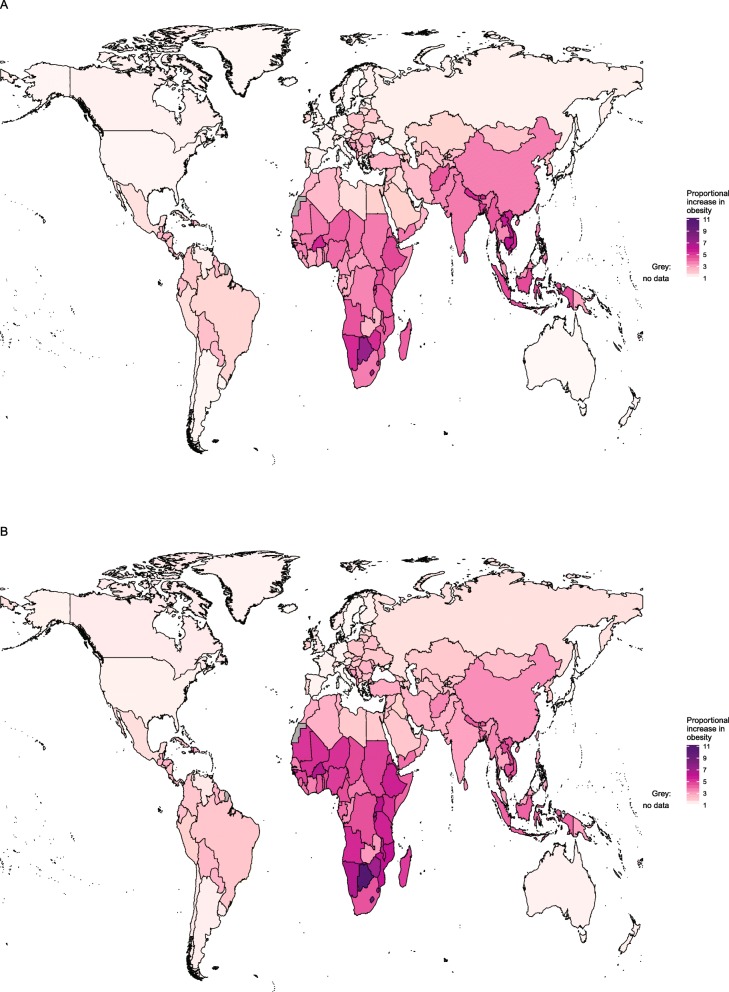
Proportional increase in obesity for girls and boys aged 5–19 years, between 1975 and 2016. Estimates of obesity for (A) girls and (B) boys were published by the Non-Communicable Diseases Risk Factor Collaboration (NCD-RisC) using the World Health Organization growth reference [47] (see Table 1)

Between 1980 and 2015, obesity prevalence in every country increased for both sexes, but there was wide variation in the extent of increase. Proportional increases per decade are shown in Fig. 7. For girls, the largest increase in obesity prevalence over time was in Botswana, where obesity increased more than seven-fold per decade, followed by Lesotho and Cambodia, where prevalence increased more than six-fold per decade. In contrast, obesity prevalence only increased by about 10% per decade in Singapore and Belgium. For boys, the proportional increases were even greater, reaching a peak in Botswana, where obesity increased more than ten-fold per decade. Again, the increase in Singapore was only approximately 10% per decade.

#### Numbers of children and adolescents with obesity

**Fig. 8:**
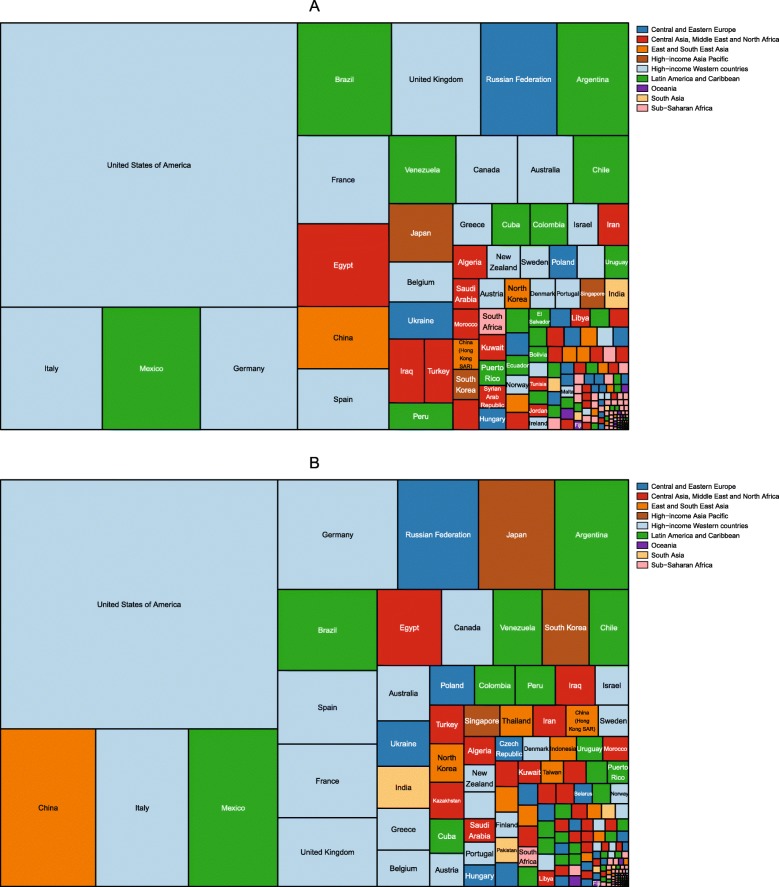
Division of the number of girls and boys aged 5–19 years with obesity in 1975, by country. Estimates of obesity for (**a**) girls and (**b**) boys were published by the Non-Communicable Diseases Risk Factor Collaboration (NCD-RisC) using the World Health Organization growth reference [47] (see Table 1)

**Fig. 9:**
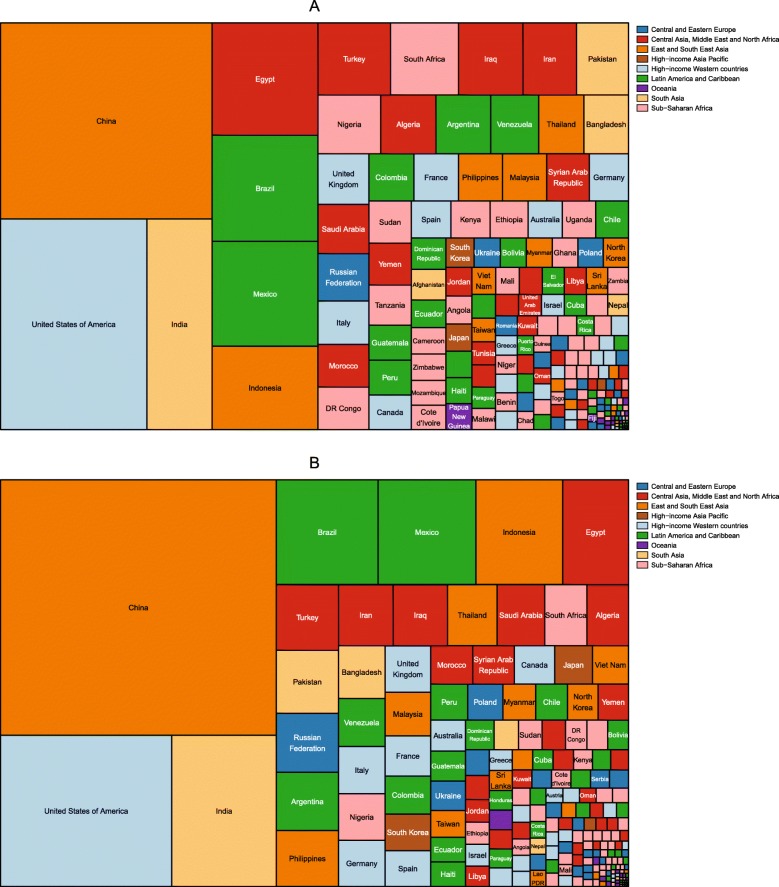
Division of the number of girls and boys aged 5–19 years with obesity in 2016, by country. Estimates of obesity for (**a**) girls and (**b**) boys were published by the Non-Communicable Diseases Risk Factor Collaboration (NCD-RisC) using the World Health Organization growth reference [47] (see Table 1)

In 1975, there were 5 million girls and 6 million boys aged 5–19 with obesity across the world. The division of these children by country is shown in Fig. 8. In 1975, the USA had the highest number of obese boys and girls aged 5–19, followed by Italy, Mexico and Germany for girls and China, Italy and Mexico for boys. By 2016, the number of children and adolescents aged 5–19 with obesity had increased to 50 million girls and 75 million boys. As shown in Fig. 9, China had the most obese boys and girls, followed by the USA and India.

#### Gender comparison

**Fig. 10:**
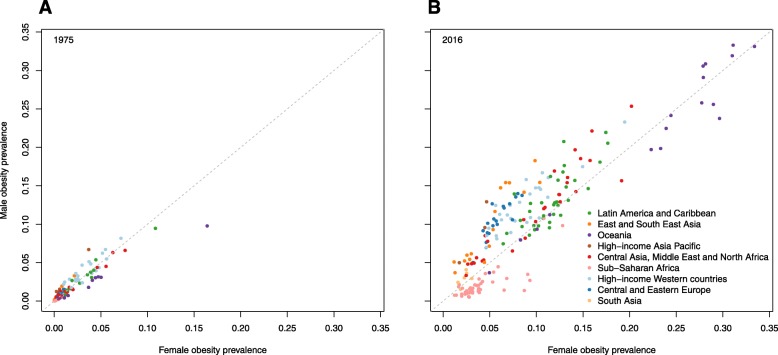
Comparison of obesity prevalence in girls and boys aged 5–19 years in 1975 and 2016. Estimates of obesity in (**a**) 1975 and (**b**) 2016 were published by the Non-Communicable Diseases Risk Factor Collaboration (NCD-RisC) using the World Health Organization growth reference [47] (see Table 1)

**Fig. 11:**
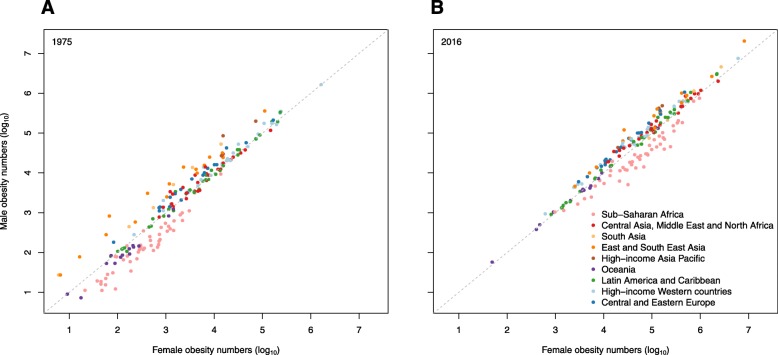
Comparison of the number of girls and boys aged 5–19 years with obesity in 1975 and 2016. Estimates of obesity for (**a**) 1975 and (**b**) 2016 were published by the Non-Communicable Diseases Risk Factor Collaboration (NCD-RisC) using the World Health Organization growth reference [47] (see Table 1)

There are clear regional differences in the relationship between obesity level and sex, as shown in Figs. 10 and 11. In 2016, the prevalence of obesity was higher in girls than boys in most countries in sub-Saharan Africa and Oceania, as well as in some other middle-income countries. In contrast, obesity was more common in boys than girls in all high-income countries, and all countries in East and Southeast Asia. Figure 11 shows the absolute numbers of girls and boys with obesity by country; again, clear regional patterns can be seen. In 2016, there were more girls than boys with obesity in almost all countries in sub-Saharan Africa and in a few other countries, but across the rest of the world, there were more boys than girls with obesity. Substantial differences in the numbers of boys and girls in the general populations of some countries can partly explain this finding. For example, in both China and India in 2016, there were 19 million more 5–19 year-old boys than girls.

## Discussion

Over the past four decades, obesity in children of all ages has increased worldwide, as it has for adults [47]. However, obesity appears to have increased more rapidly in 5–19 year olds than in younger children, with an eight-fold increase between 1975 and 2016. This contrasts with an approximate doubling in obesity rates in children aged 2–4 years between 1980 and 2015, albeit using metrics that are not directly comparable. There is heterogeneity in the levels and trends in obesity prevalence between regions and countries, depending on the stage of the global obesity epidemic they are experiencing. In particular, there has been some flattening of trends, especially among those with high socioeconomic status in high-income countries [51].

The need for high-quality and comparable data is recognised as a key component for monitoring malnutrition [3]. Data from pooled analyses allow examination of change over time and the use of standardised, comparable metrics allows trends to be benchmarked across countries. Here, we have used data from three different sources, covering different ages and countries. This limits their comparability [45–47] and, in particular, there is less standardised and comparable country-level information for children under the age of 5 years [45]. Equally, although national trends are of great interest, it is known that they mask subnational heterogeneity. Collection of disaggregated data at the subnational level and across specific groups of the population is therefore essential to identify groups that are at risk of malnutrition and to ensure progress in meeting global targets [3].

Despite overall increases in the prevalence of obesity in childhood, different forms of malnutrition coexist at global, national and subnational levels. Increases in obesity are linked to a reduction in the prevalence of children of normal weight, without there necessarily being decreases in the prevalence of children who are underweight. At a global level, the prevalence of underweight among children aged 5–19 years has remained unchanged over the past four decades [47]. Similar observations have been made in individual countries. For example, in Seychelles, only children in the upper percentiles of body mass index (BMI) have increased in weight, with little or no increase observed among those with median and low BMI [52]. More studies are needed to describe the shift in distribution of BMI over time in populations, e.g. estimates of the whole distribution to examine whether increases in BMI have occurred in all children or only in subgroups.

It is recognised that the main drivers of the current obesity epidemic are related to changing food systems and reduced physical activity [53–55], with two key features. Firstly, there is increased availability of generally inexpensive, energy-dense and ultra-processed foods and beverages. The globalisation of food supply means that it is often economically more profitable to produce and market processed, energy-dense foods than fresh ones. Recent results from the Global Burden of Disease study show that consumption of healthy foods is suboptimal, whereas that of unhealthy options exceeds recommended levels [46]. Secondly, there have been increases in the number of people with sedentary lifestyles, with high levels of physical inactivity among children [56]. As children transition through childhood and adolescence, susceptibility to the food and physical environments increases. Increasingly, children can choose the foods they eat and how much exercise they do and this has a strong impact on current and future behaviour [57–60]. This may, in part, explain the rapid increase in the prevalence of obesity in this group. Further investigation is required to explain the more rapid increase in obesity in boys, including studies of whether they are more susceptible to obesogenic pressures.

The need to improve the food environment requires governments, international organisations and other key stakeholders, including civil society and the private sector at local and global levels, to address global and local commercial determinants of obesity, including production and marketing of unhealthy, energy-dense foods and to improve availability and affordability of unprocessed healthy foods. Equally, healthy diets must be integrated with food systems in a sustainable way, such that long-term health benefits are possible [61]. A constructive dialogue with the food industry and effective regulations are needed to improve the availability of healthy foods and reduce that of unhealthy options, including prevention of unethical marketing of unhealthy foods aimed at low-income countries and other vulnerable members of the global population. The paradigm of energy imbalance (increased energy intake not balanced by energy consumption) is often used by the food industry to weaken policies aimed at tackling the use of energy-dense foods; i.e., it is argued that adequate levels of physical activity can compensate for this imbalance. Given the scale of the obesity epidemic, this argument must be viewed with scepticism. In the same vein, city and urban planners must rethink their role in society, given that current physical environments substantially restrict mobility patterns. As sedentarism becomes more common and future jobs require less activity, our children will amass a substantial cumulative burden of inactivity that will become difficult to reverse.

The effectiveness of interventions aimed at children who are overweight and obese has been widely studied [62–65]. Most interventions have targeted behavioural changes, mainly in terms of nutrition and physical activity. There is evidence that some of these interventions have been effective in schools [66, 67]. In particular, promotion of physical activity in school-based settings may be beneficial given adequate resources [68], as well as being an important component of effective overweight prevention strategies in children [66]. Efforts to promote active mobility, such as cycling lanes, are being implemented in many cities in high-income countries – and, increasingly in low or middle-income countries, including the cities adhering to the Agita Mundo Network in Latin America [69]. Recently, declines in the level of obesity among pre-school-age children have been observed in New Zealand [70], Leeds (UK) [71, 72] and Amsterdam (the Netherlands) [73]. These declines are associated with interventions aimed at supporting families and communities by creating a healthier food environment and supporting families to enforce healthy habits in children with an approach of shared responsibilities among multiple actors. This may have important implications for future trends of obesity in childhood. However, caution in causal interpretation is necessary and more evidence is needed to establish that the implemented interventions are, in fact, responsible for the observed declines in obesity in childhood [70, 71]. Equally, data and evidence on the effectiveness of community-based approaches are limited compared to school-based programmes [74, 75]. Discussions about the role of community knowledge, attitudes and awareness towards obesity in accepting policy-level solutions continue, but data supporting such evidence are lacking or show unsuccessful examples [76, 77].

Despite this work, the effects of traditional behavioural change interventions will be too small to relieve the global burden of obesity in childhood – at least in the short to medium-term [53]. Community-wide approaches matched with changes in government policies related to food reformulation, advertising and affordability are therefore also required. Policies that can achieve such changes include, among others, excise tax on beverages containing sugar, subsidies or alleviation of trade tax for producing and distributing fruit and vegetables, regulations on food labelling, restrictions on advertising of unhealthy foods and beverages, and incentives or regulations to catalyse reformulation of processed foods toward healthier composition. Other policies include giving vouchers to mothers in the USA with low incomes to purchase fruits and vegetables, low-fat or skimmed milk and whole-grain instead of refined-grain products, among other changes. This has been shown to reduce obesity rates among children aged 2–4 years [78]. However, high levels of heterogeneity in policy are observed across countries, with low and middle-income countries relying more on such approaches (and implementing them earlier) than high-income countries. For example, Mexico was one of the first countries to implement a sugar-sweetened beverage tax; 2 years after implementation, consumption had decreased by 8.2% [79]. In 2014, Chile started to implement a series of policies aimed at reducing obesity. Tax on beverages with high sugar content was increased from 13 to 18%, while tax on drinks with low or no sugar content was reduced from 13 to 10%. In 2016, a labelling system using black octagons on packaging was introduced for food and beverages that are high in sugar, calories, sodium and saturated fats. In addition, foods and beverages with such labels have been banned from schools, while marketing of these products to children under the age of 14 years is no longer allowed [80, 81]. Initial results suggest a positive impact on knowledge and awareness, reductions in consumption of unhealthy foods and a positive response from the food industry. In turn, the food industry is decreasing the amount of sugar and sodium in some food categories.

The heterogeneity in levels of obesity across the world also has important implications for global targets and goals. It is necessary to aim only for “no increase in obesity by 2025” in those regions and countries in which a clear upward trend in obesity is observed. However, much stronger political action is needed in those regions and countries where the prevalence of obesity has plateaued at high levels, to raise the priority of multi-sectoral interventions to address obesity and other chronic conditions. In general, there is a need to examine how different policy agendas [5, 6, 82–84] can be integrated and strengthened to promote healthy nutrition and regular physical activity, including preventing overweight among children, while also continuing to implement interventions against undernutrition. This will require additional efforts that should not overlook low and middle-income countries simply because some have moderate levels of obesity and high levels of undernutrition.

## Conclusions

Tackling the obesity epidemic in children will require integrated efforts across multiple sectors to provide equitable access to economic resources, education, healthy food and urban environments and to universal health coverage. Most importantly, bolder political will and accountability is needed from actors including government, civil society, academia, the private sector and other key stakeholders, to spearhead efforts to promote production of and access to a healthier environment for all.

## Data Availability

Not applicable.

## Abbreviations

IHME: Institute for Health Metrics
IOTF: International Obesity Taskforce
NCD: Non-communicable disease
WHO: World Health Organization
